# Eudaemonic Well-Being in Midlife Women: Change in and Correspondence Between Concurrent and Retrospective Reports

**DOI:** 10.1525/collabra.21433

**Published:** 2021-03-25

**Authors:** Suzanne C. Segerstrom, Tessa R. Blevins, Kate A. Leger, Rebecca G. Reed, Leslie J. Crofford

**Affiliations:** 1 Department of Psychology, University of Kentucky, Vanderbilt University Medical School; 2 Department of Psychology, University of Kentucky; Department of Psychology, Ohio State University, Vanderbilt University Medical School; 3 Department of Psychology, University of Pittsburgh, Vanderbilt University Medical School; 4 Division of Rheumatology and Immunology, Vanderbilt University Medical School

**Keywords:** eudaemonic well-being, psychological well-being, self-report, diary, longitudinal burst design

## Abstract

Concurrent and retrospective reports correspond for personality, affect, and coping. The present study described how autonomy, competence, and relatedness components of eudaemonic well-being (EWB) change over days and months and tested correspondences of daily and retrospective reports between and within people. Midlife and older (50–75 years) women (N = 200) completed online diaries daily for 1 week for 9 bursts over 2 years and answered questionnaires at the end of each burst (burst n = 1,529). Multilevel models partialed levels of variance and tested correspondence. Women varied in their daily experiences of EWB but did not vary substantially between bursts. Burst-level diary means and questionnaire responses corresponded between people, but changes within people were less strongly related. The daily, but not monthly, time scale of change is important for capturing within-person changes in EWB. Finding EWB change over months to years may depend on measurement designed to capture medium-term change.

Eudaemonia refers to well-being achieved when people are growing personally, flourishing, using their talents, engaging with their true self, and focusing on their own path, while not allowing societal norms to affect their decision making and behavior (Heintzelman, 2018; Ryan & Deci, 2001). Although dozens of constructs have been included under the umbrella of eudaemonic well-being (EWB), two frameworks defining what constitutes EWB predominate (Heintzelman, 2018). Self-determination theory (SDT) proposed that autonomy, relatedness, and competence comprise the basic human needs for growth and well-being (Martela & Sheldon, 2019; Ryan & Deci, 2001). Psychological well-being (PWB) theory proposed six components: autonomy, environmental mastery, personal growth, positive relations with others, purpose in life, and self-acceptance (Ryff & Keyes, 1995). The present study described the degree of change over days and months and tested the correspondences between autonomy, competence/mastery, and relatedness/positive relations when assessed as concurrent EWB, reported daily, and retrospective EWB, reported every 3 months, both between and within people.

Differences between concurrent and retrospective reports can arise because concurrent assessments yield reports from current experience, whereas retrospective assessments yield reports from cognitive summaries and appraisals (Bolger et al., 2003; Kahneman, 1999; Robinson & Clore, 2002; Schwarz, 2007). Over periods ranging from 2 days to 1 year, aggregate concurrent reports correlated moderately with retrospective reports for personality traits (*r* = .50), coping (*r* = .24 – .77), and emotion (*r* = .13 – .77) (Fleeson & Gallagher, 2009; Mill et al., 2016; Röcke et al., 2011; Smith et al., 1999; Stone et al., 1998; Thomas & Diener, 1990; Todd et al., 2004). The magnitude of correspondence between concurrent and retrospective reports of EWB has not been described, although experiences of autonomy, relatedness, and competence have been proposed to lead to long-term EWB (Prentice et al., 2019). Furthermore, different kinds of well-being change on different time scales; for example, day-to-day variance in affect is greater than that in life satisfaction (Willroth et al., 2020). The appropriate measurement interval and method for EWB, therefore, relies on the time scale of change as well as whether concurrent and retrospective reports correspond (Kuiper & Ryan, 2018).

In the present study, we estimated the time scale of change in EWB and linked concurrent reports of EWB in daily diaries - autonomy, relatedness, and competence - to retrospective reports of EWB. The Daily Activity and Health in the Lives of Adult Women (DAHLiA) study of midlife and older women (age 50 to 75) utilized a longitudinal burst design that included 7 daily reports followed by one retrospective report at each burst. In addition, we performed parallel analyses with hedonic well-being (HWB; e.g., affect) because it is perhaps the best-studied component of well-being with regard to the time scale of change and correspondence between concurrent and retrospective reports (cf., Willroth et al., 2020). Its inclusion served several purposes: to examine whether variability and correspondence in EWB resembled those in HWB; to allow evaluation of discriminant validity for EWB components; and to establish that the findings for HWB were similar to those in previous research, thereby situating the present study in the literature and validating comparison between HWB and EWB.

The purpose of this analysis of DAHLiA data was:

1. Estimate the levels of variance in concurrent (day, burst, and person) and retrospective (burst and person) EWB and, for the purpose of comparison, distress.
2. Link person- and burst-level concurrent EWB and, for the purpose of comparison, distress to retrospective reports. We predicted that, as was true for personality, coping, and emotion, there would be moderate correspondence.

Finally, studies with frequently repeated measurements (e.g., diary studies) and large-scale surveys necessarily rely on short forms, including single items, to alleviate participant burden and maximize retention. Construct validity of a single-item assessment with regard to a multi-item scale depends on (1) reliability of the item; (2) correspondence between the item and scale in the focal content; and (3) content coverage of the item with regard to the scale. Although adequate reliability and validity of single-item assessments have been demonstrated for other well-being constructs such as self-esteem and life satisfaction (Cheung & Lucas, 2014; Robins et al., 2001), such evidence is not readily available for single-item measures of EWB. Therefore, correspondence between concurrent and retrospective reports was contextualized with analyses examining the validity of the single items.

## Materials and Methods

### Participants

Participants were 200 women aged 50 to 75 years at study entry (M = 62 years, SD = 6.4). The sample was predominantly white (99%) with the rest African-American (1%) and generally well-educated (M = 16.7 years, SD = 2.3) in the context of a large range (12–22 years).

DAHLiA was originally powered based on the “N_effective_” (Snijders & Bosker, 1999), which considers the number of units at each level in light of the intraclass correlation (ICC) of key study variables. The target N was 300, with 2,306 bursts and 16,142 diaries after attrition, yielding .80 power to detect within-person effect sizes between *r* = .10 and *r* = .16 and between-person effects of *r* = .16. However, as the study progressed, retention and compliance was significantly higher than projected. Therefore, the target N was decreased to 200. This design change increased the smallest between-person effect size for which the study was powered at .80 (two-tailed *α* = .05) from *r* = .16 to *r* = .20.

DAHLiA was designed to examine the interactions among pain, daily activity, and psychological adjustment and well-being in midlife and older women (Judge et al., 2020; Leger et al., 2020; Salt et al., 2017; Segerstrom et al., 2016). In the United States, this demographic group is particularly likely to experience physical pain (Johannes et al., 2010). Participants from the Kentucky Women’s Health Registry aged 50–75 and living in a seven-county area in Central Kentucky were sent an email invitation to participate. Respondents were further screened for study exclusion criteria: BMI > 40; pacemaker; ongoing treatment for serious heart or other medical conditions; infectious or chronic inflammatory diseases; serious mental disorders; oral, inhaled, or injected corticosteroids in the three months prior to enrollment; severe hypertension (> 200/100mm Hg), tachycardia, bradycardia, or atrioventricular block; or any medical, neurological, or musculoskeletal condition preventing treadmill exercise (for estimation of cardiovascular fitness). Women who reported physical pain lasting more than 2 months in the registry were oversampled: 37 reported pain in more than one site, 54 reported pain in one site, and 109 reported no pain.

### Procedures

Study participation was initiated at a clinic visit in which investigators obtained informed consent, assessed physical parameters, and oriented participants to online daily diaries. Participants were sent links to 7 consecutive online daily diaries at each of 9 bursts, which occurred every 3 months over 2 years. At the end of the burst, interviewers administered questionnaire measures in person. Participants were compensated $50 at the clinic assessment and $25 at each burst, with a $25 bonus for completing all 7 diaries between 8 pm and 2 am. All procedures were approved by the University of Kentucky Institutional Review Board.

### Measures

#### Demographics.

Participants reported their age, race/ethnicity, and education at the clinic visit.

#### EWB.

Daily Autonomy was measured with the item “I felt free to decide for myself”, adapted from the Basic Need Satisfaction scale (Johnston & Finney, 2010). Competence was measured with the item “I felt competent and capable in my activities” and relatedness with the item “I felt close and connected to others” (from Reis et al., 2000). The response scale for all items ranged from 1 (not at all) to 5 (very much). Single-item measurement raises concern about reliability. The intraclass correlation (ICC) provides a lower bound on reliability if within-person variance is greater than 0. The ICC for single items captures both stability and reliability. If true scores are perfectly stable, reliability is equivalent to the ICC. If there is real change over time (i.e., instability), reliability is higher than the ICC. With ICCs for unaggregated EWB items (over time) in the range of 0.39 – 0.44 (ICC_1,_ Table 1), there was evidence for reasonable measurement reliability with these items. Furthermore, the correspondence analyses were performed using the week-long mean of diary items, which had good reliability in the range of 0.80 – 0.83 (ICC_2_, Table 1).

The Scales of Psychological Well-Being (SPWB) questionnaire with 14-item subscales (Ryff, 1989) was administered at every burst in the post-diary interview. The present analysis employed the Autonomy subscale (e.g., “My decisions are not usually influenced by what everyone else is doing”), the Environmental Mastery subscale (e.g., “I am quite good at managing the many responsibilities of my daily life”), and the Positive Relations with Others subscale (e.g., “I feel like I get a lot out of my friendships.”) The response scale ranged from 1 (strongly disagree) to 6 (strongly agree). Scale reliability was calculated both in terms of whether items covaried between people (across all bursts; R_KF_) and within people (between bursts; R_C_) (Cranford et al., 2006). All three subscales were reliable between people across bursts (Autonomy, 0.98; Environmental Mastery, 0.99; Positive Relations, 0.99). Reliability within people was lower, and the within-person reliability of Autonomy was substantially lower, suggesting that Autonomy items did not covary together as they change over time within people (Autonomy, 0.18; Environmental Mastery, 0.57; Positive Relations, 0.55).

These measures were derived from SDT and PWB perspectives, but the theoretical constructs align. For SDT and PWB respectively, autonomy is defined as the need to “self-organize experience and behavior and to have activity be concordant with one’s integrated sense of self” (Ryan & Deci, 2001, p. 213) and as “self-determining and independent; able to resist social pressures…; regulat[ing] behaviors from within; evaluates self by personal standards” (Ryff, 1989, p. 1072). Competence/mastery is defined as “a propensity to have an effect on the environment as well as to attain valued outcomes within it” (Ryan & Deci, 2001, p. 213) and as “a sense of mastery and competence in managing the environment; controls complex array of external activities; makes effective use of surrounding opportunities; able to choose or create contexts suitable to personal needs and values” (Ryff, 1989, p. 1072). Relatedness/positive relations is defined as feeling “connected to others – to love and care, and to be loved and cared for” (Ryan & Deci, 2001, p. 213) and as “has warm, satisfying, trusting relationships with others; is concerned about the welfare of others; capable of strong empathy, affection, and intimacy; understands give and take of human relationships” (Ryff, 1989, p. 1072). Although PWB dimensions can have broader theoretical scope, the core of each component of EWB aligns. Cross-sectional correlations between PWB and SDT measures among college students provide support for this assertion (Church et al., 2013; Johnston & Finney, 2010).

#### Distress.

In the diary, depression and anxiety items from the Patient-Reported Outcomes Measurement Information System (PROMIS; Cella et al., 2007) measured distress (e.g., “I felt worthless”, “I felt depressed”, “I felt uneasy”, “I felt fearful.”) Due to a clerical error, only 7 of the 8 items were included in the diary. The response scale ranged from 1 (never) to 5 (always). The negative affect items were reliable between people (0.99) and within people (across days; 0.85).

The 30-item Geriatric Depression Scale (Yesavage et al., 1982) was administered at every burst in the post-diary interview. The GDS was designed to measure affect in older adults without confounding from physical symptoms. Twenty yes-no items reflect distress (e.g., “Do you often feel downhearted and blue?”; “Do you frequently get upset over little things?”), and 10 items reflect well-being and are reversed in scoring (e.g., “Are you hopeful about the future?”; “Do you feel happy most of the time?”). Because GDS responses were dichotomous, reliability was calculated on 5 parcels of 6 items. To account for allocation variability, 150 datasets with random allocation of items to parcels were created, reliability was calculated in each dataset, and the mean and range across the 150 datasets was obtained (Sterba & MacCallum, 2010; Sterba & Rights, 2016). The GDS reliability was very high between people across bursts (mean R_KF_ = 0.99, range = 0.99 – 0.99) but lower within people between bursts (mean R_C_ = 0.48, range = 0.42 – 0.52).

### Data analysis

All measures were converted to percent of maximum possible, which facilitates comparison and interpretation of model parameters and is preferable to standardization for longitudinal models (Moeller, 2015). Sources of variance and correspondence between diary and interview measures were analyzed in multilevel models (MLM) using the lmer function of the lme4 package in R (3.6.1; see Reproducibility Statement in the supplemental online material for packages, versions, and references). Sources of variance were estimated in “empty” models using REML estimation. For diary measures, person was at the top level, burst was at the middle level, and day was at the bottom level. For questionnaire measures, person was at the top level and burst was at the bottom level. For example, for diary autonomy measured on day *i* during burst *j* for person *k*:

$$\text{autonom}y_{ijk}=\alpha_{0jk}+r_{ijk}$$

$$\alpha_{0jk}=\beta_{00k}+e_{0jk}$$

$$\beta_{00k}=\gamma_{000}+U_{00k}$$

The variances of *r*_*ijk*_, *e*_0*jk*_, and *U*_00*k*_ represent the amount of variance at the day, burst, and person levels of the model, respectively. (The model for questionnaire measures yielded only variances of *r*_*jk*_ and *U*_0*k*_.)

Another way of expressing variance components is the intraclass correlation (ICC). For questionnaire measures, which had two levels, the ICC is the ratio of person-level variance to person- and burst-level variance and represents the correlation between any two bursts within a person. For diary measures, which had three levels, there are multiple ICCs. ICC_1_ is the ratio of person-level to person-, burst-, and day-level variance and represents the correlation between any two days within a person. ICC_2_ is the ratio of person-level to person- and burst-level variance and represents the correlation between any two bursts within a person (when burst is represented as mean across days). ICC_3_ is the ratio of person- and burst-level variance to person-, burst-, and day-level variance and represents the correlation between any two days within a person and burst.

For estimating correspondence between diary and questionnaire measures, two-level MLM were constructed for burst *i* and person *j* with ML estimation and specifying a random intercept and random slope for time. A random time slope imposed a weak autoregressive covariance structure (vs. AR(1), which imposes a stronger autoregressive structure). The model with random intercept and time slope was significantly better by likelihood ratio test than a random intercept-only model, which imposed a compound symmetric covariance structure in which time interval does not affect covariance between bursts. The fixed slope for time was insubstantial and not included in the fixed portion of the model.

The explanatory diary variables (mean across all diaries [D] within a burst) were person-mean-centered so that there were two diary terms. The mean of means across all bursts reflected between-person individual differences (Level 2), and deviations from that mean at each burst reflected within-person changes (Level 1). The outcome variable was the questionnaire (Q) score at each burst. For example, for burst *i* and person *j*:

$$\text{Qautonom}y_{ij}=\beta_{0j}+\beta_{1j}*\left(\text{Dautonom}y_{ij}-\text{Dautonomy}M_{j}\right)+e_{ij}$$

$$\beta_{0j}=\gamma_{00}+\gamma_{01}\left(\text{Dautonomy}M_{j}\right)+U_{0j}$$

$$\beta_{1j}=\gamma_{10}+U_{1j}$$

The presence of a random slope (reflected by *U*_1*j*_) was tested using the likelihood ratio of models with and without random slopes for *β*_1*j*_, with mixture degrees of freedom. Models with random slopes were indicated (all *p* < .05) and included. All parameters were evaluated using Kenward-Roger degrees of freedom to correct for bias. Random effects are reported as *σ*^2^ (i.e., VAR(e_ij_)), *τ*_00_ (i.e., VAR (U_0j_)), and *τ*_11_ (i.e., VAR(U_1j_)).

Finally, the distress model that paralleled the well-being models is shown for comparison. However, GDS scores were better reflected by a zero-inflated Poisson distribution, as 28% of the bursts yielded GDS scores of 0. Therefore, a multilevel ZIP model with MLF estimation was conducted in MPlus. The results of this model were substantively the same as those of the MLM using lmer and are available in the supplemental material.

To probe the validity of the diary EWB items, first, the mean of the diary item (across all days and bursts) was correlated with each of the means of the questionnaire subscale items (across all bursts) and compared with the mean item-total correlation from the questionnaire subscale. Second, two structural equations models were fit: one with the diary item regressed on each subscale item with the weights freely estimated, and one with the diary item regressed on each subscale item with the weights constrained to be equal (Gonzalez et al., 2020). Whether this constraint compromised the model fit was tested using change in *χ*^2^ with change in degrees of freedom between the two models. Where model fit was compromised (i.e,. *p* < .05), items with high weights were allowed to have a different constrained weight *b* from the remainder of the items’ constrained weight *a*. Finally, setting *a* to 0 tested whether the diary item was related to all of the scale items or only to a few strongly related items.

## Results

### Compliance and missing data

Within diary bursts, most data were complete with 7 diary observations (85% for distress, 84% for autonomy, 83% for competence and relatedness), and almost all had 6 or 7 observations (97%). Of the 1800 possible bursts, 1529 were completed (85%). Reasons for burst-level missingness were dropout (too busy, N = 8; moved, N = 4; illness, N = 7; schedule, N = 2), lost to follow-up (N = 20), and death (N = 1).

### Variance components

**Figure 1.**
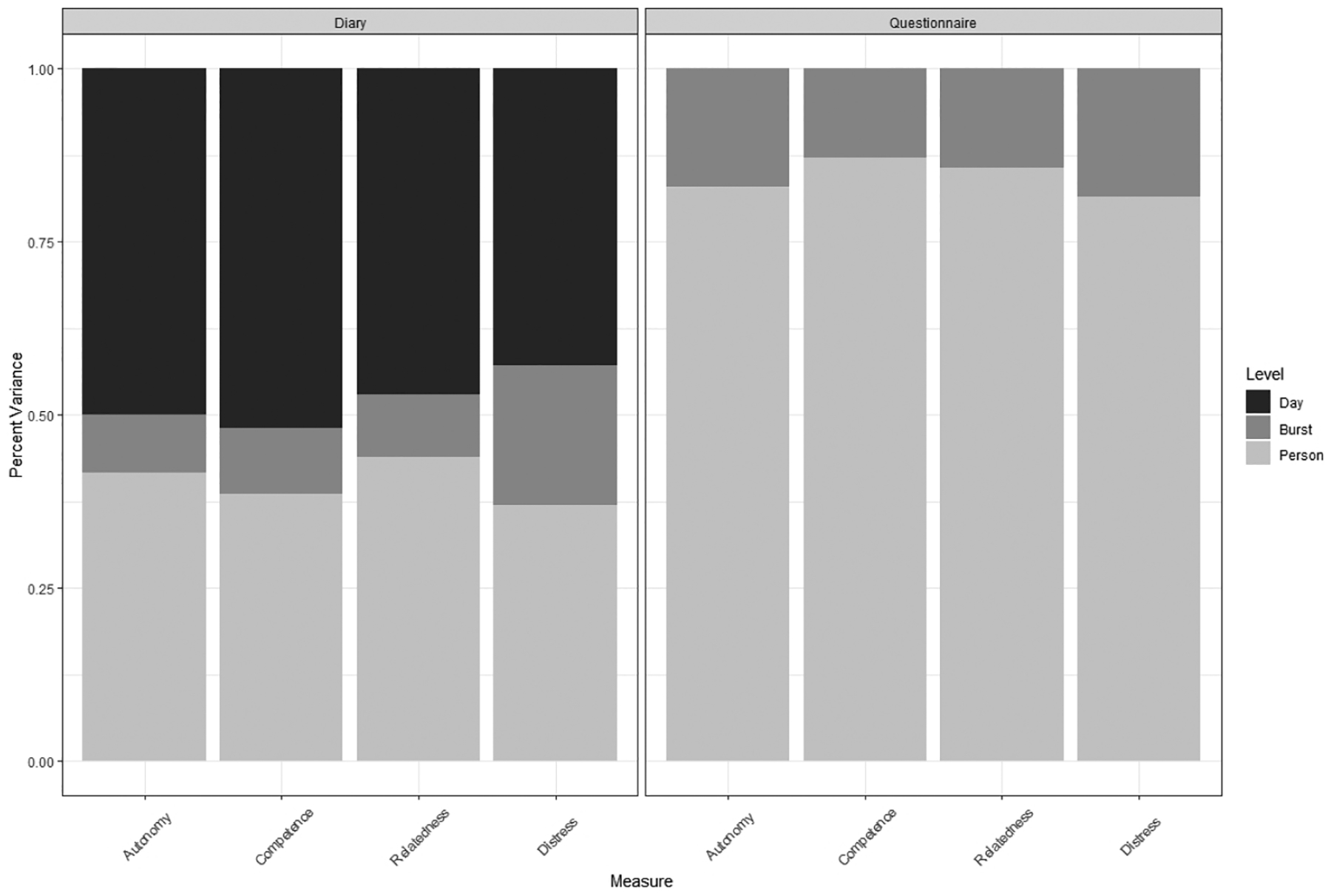
Levels of variance in the daily diary and burst-level questionnaire components of autonomy, relatedness, competence, and distress.

Figure 1 shows the sources of variance in the daily diary and burst-level questionnaire components of autonomy, relatedness, competence, and distress. When measured daily, variance for all four constructs was primarily found at the day and person levels. Note that the day level contains both day variance and measurement error for the single items in the diary. However, correction for unreliability (see ICC results below) suggests that most of the variance at the day level was true change. EWB varied little at the burst level. Distress varied more from burst to burst, particularly in the daily diary. Therefore, women varied substantially from day to day in their experiences of EWB, and they also varied substantially from each other. However, their levels of diary well-being averaged across a week-long burst did not change substantially between bursts. When measured retrospectively at end-of-burst, questionnaire variance for all four constructs was primarily at the person level; women varied substantially from each other but did not change substantially between bursts.

Table 1 shows the descriptive statistics for person-level variables (mean across all days and/or bursts), ICCs, and correlations among the person-level variables. For diary variables, the correlation between any two days within a person (ICC_1_) was moderate at ~ *r* = 0.40. Assuming single item reliability of 0.50, reliability-adjusted ICC_1_s were 0.55 for autonomy (vs. 0.42 uncorrected), 0.52 for competence (vs. 0.39), and 0.58 for relatedness (vs. 0.44) (Wilms et al., 2020). It is therefore reasonable to conclude that approximately half of the variance was due to person, with the remainder due to changes among bursts and days.

For diary variables, the correlation between any two days within a burst (ICC_3_) was also modest at ~ *r* = 0.50, and not much higher than the ICC_1_, a result expectable from the small amount of burst variance (see Figure 1). For both diary and questionnaire variables, the correlation between any two bursts within a person (ICC_2_ for diary, ICC_1_ for questionnaire) was high at *r* > .80 (except for diary distress, *r* = 0.65).

### Correspondence between concurrent and retrospective well-being: Between- and within-person

Table 1 correlations provide the first evidence for correspondence and convergent validity. Diary and questionnaire components of EWB were positively correlated with each other at the between-person (mean across all assessments) and within-person (using person-mean-centered values to remove between-person variance) levels. Between people, competence from diary and questionnaire was most highly correlated across people (*r* = .74), and autonomy was least highly correlated (*r* = 0.34). At the burst level, there was less evidence for correspondence. Within people, changes in diary and questionnaire autonomy were uncorrelated across bursts (*r* = .00). There were small correlations for competence and relatedness (*r* = .15 and .11, respectively), and the largest correlation was for distress (*r* = .20).

To further test correspondence at between-person and within-person levels, burst-level, concurrent diary means were used as explanatory variables with retrospective EWB (SPWB questionnaire) as outcome variables in multilevel models. These models provide inferential statistics for within-person effects in clustered data, which correlations cannot, and also estimate variances and covariances for random effects. Because both variables were expressed as percent of maximum possible (POMP), the estimates reflect the POMP difference in the SPWB questionnaire for each POMP within-person change or between-person difference in the diary mean. Table 2 gives the estimates (*γ*_*10*_ for within-person effects, *γ*_*01*_ for between-person effects in the equations above) with their 95% confidence intervals and *p* values. All the relationships between diary reports and questionnaire reports were statistically significant, except for burst-level changes in autonomy. The magnitude of these relationships varied; in particular, between-person individual differences corresponded more closely than did within-person changes.

### Correspondence between concurrent and retrospective well-being: Validity evidence

Low correlations between diary and questionnaire could reflect poor correspondence between daily and retrospective reports but also could reflect poor convergent validity either because the two are measuring different constructs (low correlations between the diary item and the questionnaire items; Strauss & Smith, 2009) or because the diary item does not have the breadth of coverage of the questionnaire (the diary item correlates highly with some questionnaire items but does not correlate with others; Cheung & Lucas, 2014). Table 3 shows the between-person correlations between the diary item and each of the questionnaire items.

For autonomy, correlations between the diary and the questionnaire items were lower than the mean item-total correlation within the questionnaire. To gauge overall coverage, a structural equations model in which the diary item was regressed on each questionnaire item with weights freely estimated was compared with a model in which all weights were constrained to be equal. Although the construct validity for autonomy was low, constraining all weights to be equal did not significantly compromise model fit (*χ*^2^ (13) = 17.3, *p* = .19). This pattern generally indicates low construct convergence but equal breadth of coverage.

Construct validity for competence was better, with diary correlations resembling the mean item-total correlation. However, constraining all weights to be equal significantly compromised model fit (*χ*^2^ (13) = 24.4, p = .029). The weights for two questionnaire items, #1 “In general, I feel I am in charge of the situation in which I live” and #4 “I am quite good at managing the many responsibilities of my daily life”, were higher than the rest. Constraining these two weights to be equal but different from the remainder of the weights (which were all constrained to be equal) did not significantly compromise model fit compared with the freely estimated model (*χ*^2^ (12) = 15.2, *p* = .23) and improved fit compared with the fully constrained model (*χ*^2^ (1) = 9.0, *p* < .0001). Constraining the remaining weights to 0 compromised model fit compared with this third model (*χ*^2^ (1) = 17.9, *p* = .0027). Although the weights were smaller with the remainder of items, they were necessary. This pattern generally indicates good construct convergence for the diary item, with coverage favoring some content but spread across the questionnaire items.

Correspondence for relatedness was also generally good, with correlations approaching the mean item-total correlation. Constraining all weights to be equal did not significantly compromise model fit (*χ*^2^ (13) = 22.0, p = .056). However, the weights for two questionnaire items, #2 [reversed] “Maintaining close relationships has been difficult and frustrating for me” and #3 [reversed] “I often feel lonely because I have few close friends with whom to share my concerns”, were higher than the rest. Constraining these two weights to be equal but different from the remainder of the weights (which were all constrained to be equal) did not significantly compromise model fit compared with the freely estimated model (*χ*^2^ (12) = 16.1, *p* = .18) and improved fit compared with the fully constrained model (*χ*^2^ (1) = 5.8, *p* = .016). Constraining the remaining weights to 0 compromised model fit compared with this third model (*χ*^2^ (1) = 7.5, *p* = .0062). Although the weights were smaller with the remainder of items, they were necessary. This pattern generally indicates good convergent validity, with coverage favoring some content but spread across the questionnaire items.

Table 1 correlations also speak to discriminant validity. Diary components of EWB were negatively correlated with distress between people, and correlations among components were larger than those with distress (all *p* < .05, except the difference between *r*_comp,rel_ and *r*_comp,distress_, *p* = .056). Therefore, there is evidence for discriminant validity of EWB components with regard to distress for the diary items. For questionnaire components, all components correlated negatively with distress. However, only for the difference between *r*_auto,comp_ and *r*_auto,distress_ was the correlation between components larger than that with distress. In the rest of cases, the difference was not statistically significant, or the magnitude of the correlation was significantly larger between the EWB component and distress than the EWB component and another component. Therefore, although correlations with distress were in the expected direction, there was more evidence against discriminant validity in the questionnaire than for it when the magnitude of the relationships was considered.

Among EWB components across diary and questionnaire, evidence was worst for autonomy, where diary autonomy correlated significantly more highly with questionnaire competence than with questionnaire autonomy between people, and the same pattern was true within people, albeit all within-person discriminant validity correlations were small. Questionnaire autonomy was also not more highly correlated with diary autonomy than diary competence. Discriminant validity of competence and relatedness were better, as the convergent correlations were generally higher than the discriminant correlations. However, between people, although diary relatedness was more highly correlated with questionnaire relatedness than was diary competence, the relationship between diary relatedness and questionnaire relatedness was not significantly different from the relationship with questionnaire competence. In addition, the high correlations among diary EWB measures also speak to a lack of discrimination among them.

## Discussion

When midlife and older women reported their EWB daily for 7 days over 9 bursts at 3-month intervals, the vast majority of the variance was associated with daily changes and between-person differences, and a minority of variance was associated with changes between bursts (Figure 1). Intraclass correlations between days within a person were moderate (ICC_1_ across all days and bursts, ICC_3_ across all days within bursts), and correlations across bursts were high (ICC_2_; Table 1). When EWB was reported retrospectively at each burst, the results were similar: variance in between-person differences was larger than that in burst-level change (Figure 1), and correlations across bursts were high (ICC_1_; Table 1).

The time scale of EWB change is important for studying within-person phenomena (Kuiper & Ryan, 2018). Among midlife and older women, there was little change in autonomy, competence, and relatedness at the time scale of months. Therefore, within-person psychological, behavioral, and health correlations with EWB may not be observed at that time scale. One reason may be that the SPWB measures of autonomy, competence, and relatedness all had poor reliability for change (Autonomy, 0.18; Environmental Mastery, 0.57; Positive Relations, 0.43; see Method), particularly Autonomy, which was the only component uncorrelated with changes in diary EWB. The SPWB were designed to capture between-person differences and not within-person change (Ryff, 1989). As a result, SPWB total and subscale scores are impressively stable. Over the course of several years among older men and women, the ICC_1_ for the total SPWB score was .83, and the ICC_1_s for the autonomy and mastery scales were .67 and .64, respectively (Segerstrom et al., 2015; Wettstein et al., 2015). Over a broader age range, the test-retest correlation for the SPWB over 9 years was .63 (Rush et al., 2019). These estimates are not substantially lower than the ICC obtained over 18 days among older adults, suggesting little decay in stability with increasing time (total, 0.64; autonomy, 0.49; mastery, 0.61; positive relations, 0.76; Saajanaho et al., 2020). A measure of EWB specifically designed to find more evidence of change over months to years may indeed do so. On the other hand, the daily measure also did not change much over months. It did have substantial change at the time scale of days, where daily activities and stressors may have contributed to daily changes (Reis et al., 2000). These findings suggest that not to measure EWB daily is to miss an important level of experience.

For the most part, EWB and distress had similar variance structures. Compared with EWB, diary distress had more burst-level variance (Figure 1; ICC_1_ < ICC_3_ in Table 1), but questionnaire distress had similar burst-level variance (Figure 1; ICC_1_ in Table 1). The higher amount of burst-level, within-person variance in diary distress potentially explains the larger within-person correspondence between diary and questionnaire (Table 2). This result also suggests that women were in fact experiencing psychological change between bursts; they were simply not changing as much in EWB.

At the person level, correlations between aggregated concurrent and retrospective EWB over 1 week resembled those for other psychological variables over similar time frames. Over 1 week, concurrent and retrospective accounts of coping correlated *r* = .50 – .77 (Smith et al., 1999). Over 2 weeks, concurrent and retrospective accounts of affect correlated *r* = .31 – .47 (Mill et al., 2016). Over 10 days among older, nontraditional students, concurrent accounts of behavior related to personality traits correlated *r* = .29 – .52 with self-reported personality traits (Fleeson & Gallagher, 2009, Study 12). In the present study, correlations for competence (*r* = .74) were at the high end of these ranges, and autonomy (*r* = .34), at the low end. It may be easier to recollect specific instances of competence and relatedness, but harder to recollect specific instances of autonomous action. Additionally, instances of low autonomy might be infrequent in this largely white and well-educated sample, whose resources might have enabled more autonomy. However, between-person standard deviations were similar for all three EWB components, and within-person standard deviations had similar medians and ranges, indicating no restriction of range between or within people. Autonomy also had the poorest convergent and discriminant validity, particularly with regard to competence. Although the theoretical definitions for SDT and PWB autonomy correspond, the poorer reliability for change in SPWB autonomy and the lower correlations with concurrent reports of autonomy indicate a need for further investigation into meaning and measurement in this construct.

By contrast, there was more evidence for convergent validity for competence and relatedness. When averaged across all assessments to maximize reliability, the single competence item correlated with the SPWB items at or above the mean item-total correlation among the SPWB items. There was evidence that the item did not relate to all of the content of the scale to the same degree in that two items regarding “the situation in which I live” and “my daily life” were more strongly related than the remainder of the items. These items seem to be more global assessments of competence; however, the single item provided content coverage beyond these two SPWB items. The single relatedness item was correlated with the SPWB items at or below the mean item-total correlation among the SPWB items. Although the model in which the item was related to all SPWB items equally was acceptable, there was evidence that the item did not capture all of the content of the scale to the same degree in that two items regarding “close relationships” and “close friends” were more strongly related than the remainder of the items. This is sensible, given that the diary item refers to feeling “close”. However, the item did provide content coverage beyond these two items. Therefore, correspondence analyses for competence and relatedness can be attributed at least in part to similarities and differences between retrospective and concurrent reports.

There was a surprising proportion of women, about 10% of the sample, who reported maximum EWB or minimum distress across all diaries. One possibility is that this sub-sample experienced very high well-being on some dimension across the entire study. Another, however, is that these women are psychologically different from those who did not report perfect well-being. Very low scores (0–1) on the Beck Depression Inventory, compared with low scores, are associated with efforts to present oneself in a favorable light (Clark et al., 1998; Joiner et al., 2000). However, most women did not report perfect scores for each of the 4 constructs, suggesting that they differentiated between the constructs and were perhaps not globally engaging in impression management (perfect on 4, n = 3; on 3, n = 6; on 2, n = 14; on 1, n = 22). Repeating the correspondence analyses without scores that were perfect across the study did not substantively change the results reported in Table 2 (see supplemental material).

Methodological strengths of DAHLiA include the burst design, which permitted measurement of well-being at multiple levels as well as variance partition at short-, medium-, and long-term intervals. The study had excellent compliance confirmed by time stamp in the online diary. The study was also powered to detect small to medium effects. However, its greatest methodological weakness is generalizability. Because the study focused on a group likely to experience physical pain (midlife and older women), these results do not necessarily generalize to younger people, men, or both. The sample was also primarily white (99%), which is generally characteristic of the population of the sampling area (94% white), but further limits generalizability. In addition, the older age, high education, and low racial diversity of the present sample might have restricted range in EWB. EWB and education are positively correlated, as are EWB and age (Ryff & Singer, 2008). In one study, minority status positively correlated with EWB (Ryff et al., 2003). Therefore, a more diverse sample might find different distributions of variance in EWB and distress. Furthermore, random slopes for correlated change in EWB components indicated individual differences in correspondence between concurrent and retrospective reports even within this homogeneous sample. These individual differences need to be explained theoretically and empirically (cf. Willroth et al., 2020). Extending these findings in more diverse samples will be useful to understanding when and how EWB differs between people and changes within people.

## Conclusion

Personality, affect, and coping have all exhibited modest correspondence between daily or momentary concurrent reports and global, retrospective reports. The present study extended this framework to EWB, obtaining similar results. In addition to testing correspondence, the results point to the presence of short- but not medium-term change in EWB, need for development of better measures for medium-term change in EWB and for autonomy, and extension of these findings to more diverse samples.

## Supplementary Material

Supp material

## Figures and Tables

**Table 1.** Intraclass correlations for diary and questionnaire variables and between-person correlations with their 95% confidence intervals below the diagonal. Within-person, burst-level correlations are above the diagonal; no confidence intervals are provided because they are not valid for these within-person correlations. Bold values indicate diary-questionnaire correspondence.

	ICC_1_	ICC_2/1_	ICC_3_	1. DA	2. DC	3. DR	4. DD	5. QA	6. QC.78	7. QR	8. QD
1. Diary Autonomy (DA)	0.42	0.83	0.50	-	0.56	0.39	−0.32	**0.00**	0.13	0.00	−0.09
2. Diary Competence (DC)	0.39	0.80	0.48	0.82 [.77, .86]	_-_	0.49	−0.38	0.02	**0.15**	0.02	−0.14
3. Diary Relatedness (DR)	0.44	0.83	0.53	0.61 [.51, .69]	0.69 [.61, .76]	_-_	−0.34	0.04	0.16	**0.11**	−0.09
4. Diary Distress (DD)	0.37	0.65	0.57	−0.49 [−.59, −.38]	−0.56 [−.65, −.46]	−0.51 [−.61, −.40]	_-_	−0.04	−0.21	−0.07	**0.20**
5. Questionnaire Autonomy (QA)	-	0.83	_-_	**0.34** [.21, .46]	0.39^1^ [.27, .50]	0.21 [.07, .34]	−0.27 [−.40, −.14]	_-_	0.21	0.20	−0.06
6. Questionnaire Competence (QC)	-	0.87	_-_	0.60^2^ [.50, .68]	**0.74** [.67, .80]	0.55^3^ [.44, .64]	−0.57 [−.66, −.47]	0.47 [.36, .57]	_-_	0.37	−0.32
7. Questionnaire Relatedness (QR)	_-_	0.83	_-_	0.30^4^ [.16, .42]	0.44^5^ [.32, .55]	**0.56** [.46, .65]	−0.31 [−.43, −.18]	0.28 [.15, .41]	0.55 [.45, .64]	_-_	−0.22
8. Questionnaire Distress (QD)	-	0.81	-	−0.47 [−.57, −.36]	−0.59 [−.67, −.49]	−0.52 [−.61, −.41]	**0.60** [.51, .68]	−0.24 [−.37, −.10]	−0.78 [−.83, −.72]	−0.53 [−.62, −.42]	-

ICC_1_ = Intraclass correlation between any two days in the diary or any two bursts in the questionnaire; ICC_2_ = Intraclass correlation between any two bursts (mean across all days) in the diary; ICC_3_ = Intraclass correlation between any two days within a burst in the diary. Differences between correlations from Lee & Preacher (2013).

1 *r*_QA,DA_ vs. *r*_QA, DC_, t(197) = 1.27, *p* = .10

2 *r*_QA,DA_ vs. *r*_QC, DA_, t(197) = 4.45, *p* = .00001

3 *r*_QR,DR_ vs. *r*_QC,DR_, t(197) = 2.59, *p* = .005

4 *r*_QA,DA_ vs. *r*_QR,DA_, t(197) = 2.01, *p* = .023

5 *r*_QR,DR_ vs. *r*_QR,DC_, t(197) = 0.19, *p* = .43

**Table 2.** Results of multilevel models testing correspondence between questionnaire and diary well-being.

	Questionnaire Autonomy			Questionnaire Competence			Questionnaire Relatedness			Questionnaire Distress		
*Predictors*	*Estimates*	*CI*	*p*	*Estimates*	*CI*	*p*	*Estimates*	*CI*	*p*	*Estimates*	*CI*	*p*
(Intercept)	69.12	67.50, 70.74	<0.001	74.91	73.70, 76.12	<0.001	76.53	75.16, 77.89	<0.001	9.17	8.07, 10.28	<0.001
D Mean	0.30	0.19, 0.41	<0.001	0.78	0.68, 0.88	<0.001	0.39	0.31, 0.47	<0.001	1.01	0.83, 1.19	<0.001
D Change	0.01	−0.02, 0.04	0.631	0.08	0.04, 0.12	<0.001	0.05	0.03, 0.08	<0.001	0.22	0.14, 0.30	<0.001
*Random Effect Estimates*												
σ^2^		26.26			20.29			18			18.72	
τ_00_ Intercept		132.48			83.72			102.25			69.12	
τ_11_ D change slope		0.00			0.02			0.00			0.05	
τ_11_Time slope		0.65			0.34			0.59			0.24	
ρ_01_ Intercept-D change		1.00			−0.08			−0.97			0.16	
ρ_01_ Intercept-Time		−0.12			−0.40			−0.30			−0.38	

CI = 95% confidence interval; D = diary.

**Table 3.** Between-person correlations between diary and questionnaire items with their 95% confidence intervals and the mean questionnaire item-total correlation for comparison

Questionnaire Subscale Item Number	Diary Autonomy “I felt free to decide for myself”	Diary Competence “I felt competent and capable in my activities”	Diary Relatedness “I felt close and connected to others”
1	.20 [.06, .33]	.66 [.57, .73]	.46 [.34, .56]
2	.23 [.09, .36]	.58 [.48, .67]	.51 [.40, .61]
3	.26 [.13, .38]	.36 [.23, .48]	.52 [.41, .62]
4	.36 [.23, .47]	.71 [.64, .78]	.42 [.30, .53]
5	.26 [.13, .39]	.57 [.47, .66]	.31 [.18, .43]
6	.24 [.11, .37]	.48 [.36, .58]	.50 [.39, .60]
7	.24 [.10, .37]	.45 [.33, .55]	.43 [.31, .54]
8	.17 [.03, .30]	.57 [.47, .66]	.43 [.31, .54]
9	.32 [.19, .44]	.63 [.54, .71]	.46 [.34, .56]
10	.27 [.14, .40]	.60 [.50, .68]	.48 [.37, .58]
11	.21 [.08, .34]	.62 [.53, .70]	.45 [.33, .55]
12	.24 [.11, .37]	.44 [.32, .54]	.35 [.22, .47]
13	.31 [.18, .43]	.61 [.52, .69]	.32 [.19, .44]
14	.33 [.20, .45]	.51 [.40, .60]	.29 [.16, .41]
Mean Item-Total Correlation	.53	.52	.55

## References

[R1] Bolger N, Davis A, & Rafaeli E (2003). Diary methods: Capturing life as it is lived. Annual Review of Psychology, 54(1), 579–616. 10.1146/annurev.psych.54.101601.145030 12499517

[R2] Cella D, Yount S, Rothrock N, Gershon R, Cook K, Reeve B, Ader D, Fries JF, Bruce B, & Rose M (2007). The Patient-Reported Outcomes Measurement Information System (PROMIS). Medical Care, 45(5), S3–S11. 10.1097/01.mlr.0000258615.42478.55 PMC2829758 17443116

[R3] Cheung F, & Lucas RE (2014). Assessing the validity of single-item life satisfaction measures: Results from three large samples. Quality of Life Research, 23(10), 2809–2818. 10.1007/s11136-014-0726-4 24890827 PMC4221492

[R4] Church AT, Katigbak MS, Ching CM, Zhang H, Shen J, Arias RM, Rincon BC, Morio H, Tanaka-Matsumi J, Takaoka S, Mastor KA, Roslan NA, Ibáñez-Reyes J, Vargas-Flores J. de J., Locke KD, Reyes JAS, Wenmei S, Ortiz FA, & Alvarez JM (2013). Within-individual variability in self-concepts and personality states: Applying density distribution and situation-behavior approaches across cultures. Journal of Research in Personality, 47(6), 922–935. 10.1016/j.jrp.2013.09.002

[R5] Clark DA, Crewdson N, & Purdon C (1998). No worries, no cares: An investigation into self-reported “nondistress” in college students. Cognitive Therapy and Research, 22, 209–224.

[R6] Cranford JA, Shrout PE, Iida M, Rafaeli E, Yip T, & Bolger N (2006). A procedure for evaluating sensitivity to within-person change: Can mood measures in diary studies detect change reliably? Personality and Social Psychology Bulletin, 32(7), 917–929. 10.1177/0146167206287721 16738025 PMC2414486

[R7] Fleeson W, & Gallagher P (2009). The implications of Big Five standing for the distribution of trait manifestation in behavior: Fifteen experience-sampling studies and a meta-analysis. Journal of Personality and Social Psychology, 97(6), 1097–1114. 10.1037/a0016786 19968421 PMC2791901

[R8] Gonzalez O, MacKinnon DP, & Muniz FB (2020). Extrinsic convergent validity evidence to prevent jingle and jangle fallacies. Multivariate Behavioral Research. Published ahead of print. 10.1080/00273171.2019.1707061 PMC7369230 31958017

[R9] Heintzelman SJ (2018). Eudaimonia in the contemporary science of subjective well-being: Psychological well-being, self-determination, and meaning in life. In Diener E, Oishi S, & Tay L (Eds.), Handbook of Well-Being. DEF Publishers. https://doi.org/DOI:nobascholar.com

[R10] Johannes CB, Le TK, Zhou X, Johnston JA, & Dworkin RH (2010). The prevalence of chronic pain in United States adults: Results of an Internet-based survey. The Journal of Pain, 11(11), 1230–1239. 10.1016/j.jpain.2010.07.002 20797916

[R11] Johnston MM, & Finney SJ (2010). Measuring basic needs satisfaction: Evaluating previous research and conducting new psychometric evaluations of the Basic Needs Satisfaction in General Scale. Contemporary Educational Psychology, 35(4), 280–296. 10.1016/j.cedpsych.2010.04.003

[R12] Joiner TE, Schmidt NB, Lerew DR, Cook JH, Gencoz T, & Gencoz F (2000). Differential roles of depressive and anxious symptoms and gender in defensiveness. Journal of Personality Assessment, 75(2), 200–211. 10.1207/s15327752jpa7502_2 11020139

[R13] Judge ST, Clasey JL, Crofford LJ, & Segerstrom SC (2020). Optimism and pain interference in aging women. Annals of Behavioral Medicine, 54(3), 202–212. 10.1093/abm/kaz040 31634392 PMC7309584

[R14] Kahneman D (1999). Objective happiness. In Kahneman D, Diener E, & Schwarz N (Eds.), Well-being: The foundations of hedonic psychology (pp. 3–25). Russell Sage Foundation.

[R15] Kuiper RM, & Ryan O (2018). Drawing conclusions from cross-lagged relationships: Re-considering the role of the time-interval. Structural Equation Modeling: A Multidisciplinary Journal, 25(5), 809–823. 10.1080/10705511.2018.1431046

[R16] Lee IA, & Preacher KJ (2013). Calculation for the test of the difference between two dependent correlations with one variable in common [Computer software]. http://quantpsy.org

[R17] Leger KA, Blevins TR, Crofford LJ, & Segerstrom SC (2020). Mean levels and variability in psychological well-being and associations with sleep in midlife and older women. Annals of Behavioral Medicine. Published online ahead of print. 10.1093/abm/kaaa069 PMC8427537 32857116

[R18] Martela F, & Sheldon KM (2019). Clarifying the concept of well-being: Psychological need satisfaction as the common core connecting eudaimonic and subjective well-being. Review of General Psychology, 23(4), 458–474. 10.1177/1089268019880886

[R19] Mill A, Realo A, & Allik J (2016). Retrospective ratings of emotions: The effects of age, daily tiredness, and personality. Frontiers in Psychology, 6. 10.3389/fpsyg.2015.02020 PMC4707248 26793142

[R20] Moeller J (2015). A word on standardization in longitudinal studies: Don’t. Frontiers in Psychology, 6. 10.3389/fpsyg.2015.01389 PMC4569815 26441764

[R21] Prentice M, Jayawickreme E, & Fleeson W (2019). Integrating whole trait theory and self-determination theory. Journal of Personality, 87(1), 56–69. 10.1111/jopy.12417 29999534

[R22] Reis HT, Sheldon KM, Gable SL, Roscoe J, & Ryan RM (2000). Daily well-being: The role of autonomy, competence, and relatedness. Personality and Social Psychology Bulletin, 26(4), 419–435. 10.1177/0146167200266002

[R23] Robins RW, Hendin HM, & Trzesniewski KH (2001). Measuring global self-esteem: Construct validation of a single-item measure and the Rosenberg Self-Esteem Scale. Personality and Social Psychology Bulletin, 27(2), 151–161. 10.1177/0146167201272002

[R24] Robinson MD, & Clore GL (2002). Belief and feeling: Evidence for an accessibility model of emotional self-report. Psychological Bulletin, 128(6), 934–960. 10.1037/0033-2909.128.6.934 12405138

[R25] Röcke C, Hoppmann CA, & Klumb PL (2011). Correspondence between retrospective and momentary ratings of positive and negative affect in old age: Findings from a one-year measurement burst design. The Journals of Gerontology: Series B, 66B(4), 411–415. 10.1093/geronb/gbr024 21444586

[R26] Rush J, Rast P, Almeida DM, & Hofer SM (2019). Modeling long-term changes in daily within-person associations: An application of multilevel SEM. Psychology and Aging, 34(2), 163–176. 10.1037/pag0000331 30730161 PMC6424492

[R27] Ryan RM, & Deci EL (2001). On happiness and human potentials: A review of research on hedonic and eudaimonic well-being. Annual Review of Psychology, 52(1), 141–166. 10.1146/annurev.psych.52.1.141 11148302

[R28] Ryff CD (1989). Happiness is everything, or is it? Explorations on the meaning of psychological well-being. Journal of Personality and Social Psychology, 57(6), 1069–1081. 10.1037/0022-3514.57.6.1069

[R29] Ryff CD, Keyes CL, & Hughes DL (2003). Status inequalities, perceived discrimination, and eudaimonic well-being: Do the challenges of minority life hone purpose and growth? Journal of Health and Social Behavior, 44(3), 275–291. 14582308

[R30] Ryff CD, & Keyes CLM (1995). The structure of psychological well-being revisited. Journal of Personality and Social Psychology, 69(4), 719–727. 10.1037/0022-3514.69.4.719 7473027

[R31] Ryff CD, & Singer BH (2008). Know thyself and become what you are: A eudaimonic approach to psychological well-being. Journal of Happiness Studies, 9(1), 13–39. 10.1007/s10902-006-9019-0

[R32] Saajanaho M, Kokko K, Pynnönen K, Tourunen A, Törmäkangas T, Portegijs E, & Rantanen T (2020). The Scales of Psychological Well-Being–a validation, usability and test–retest study among community-dwelling older people in Finland. Aging & Mental Health. Published ahead of print. 10.1080/13607863.2020.1725801 32052647

[R33] Salt E, Crofford LJ, & Segerstrom S (2017). The mediating and moderating effect of volunteering on pain and depression, life purpose, well-being, and physical activity. Pain Management Nursing, 18(4), 243–249. 10.1016/j.pmn.2017.04.004 28601476 PMC7251856

[R34] Schwarz N (2007). Retrospective and concurrent self-reports: The rationale for real-time data capture. In Shiffman SS, Atienza A, & Nebeling L (Eds.), The science of real-time data capture: Self-reports in health research (pp. 11–26). Oxford University Press.

[R35] Segerstrom SC, Eisenlohr-Moul TA, Evans DR, & Ram N (2015). Repetitive thought dimensions, psychological well-being, and perceived growth in older adults: A multilevel, prospective study. Anxiety, Stress, & Coping, 28(3), 287–302. 10.1080/10615806.2014.947285 PMC4334760 25055116

[R36] Segerstrom SC, Jones AC, Scott AB, & Crofford LJ (2016). Daily goals and psychological well-being in midlife and older women: Physical pain interacts with goal conflict. Research in Human Development, 13(4), 328–341. 10.1080/15427609.2016.1234306 28603467 PMC5464603

[R37] Smith RE, Leffingwell TR, & Ptacek JT (1999). Can people remember how they coped? Factors associated with discordance between same-day and retrospective reports. Journal of Personality and Social Psychology, 76(6), 1050–1061. 10.1037/0022-3514.76.6.1050

[R38] Snijders TA, & Bosker RJ (1999). Multilevel analysis: An introduction to basic and advanced multilevel modeling. Sage.

[R39] Sterba SK, & MacCallum RC (2010). Variability in parameter estimates and model fit across repeated allocations of items to parcels. Multivariate Behavioral Research, 45(2), 322–358. 10.1080/00273171003680302 26760288

[R40] Sterba SK, & Rights JD (2016). Accounting for parcel-allocation variability in practice: Combining sources of uncertainty and choosing the number of allocations. Multivariate Behavioral Research, 51(2–3), 296–313. 10.1080/00273171.2016.1144502 27054282

[R41] Stone AA, Schwartz JE, Neale JM, Shiffman S, Marco CA, Hickcox M, Paty J, Porter LS, & Cruise LJ (1998). A comparison of coping assessed by ecological momentary assessment and retrospective recall. Journal of Personality and Social Psychology, 74(6), 1670–1680. 10.1037/0022-3514.74.6.1670 9654765

[R42] Strauss ME, & Smith GT (2009). Construct validity: Advances in theory and methodology. Annual Review of Clinical Psychology, 5(1), 1–25. 10.1146/annurev.clinpsy.032408.153639 PMC2739261 19086835

[R43] Thomas DL, & Diener E (1990). Memory accuracy in the recall of emotions. Journal of Personality and Social Psychology, 59(2), 291–297. 10.1037/0022-3514.59.2.291

[R44] Todd M, Tennen H, Carney MA, Armeli S, & Affleck G (2004). Do we know how we cope? Relating daily coping reports to global and time-limited retrospective assessments. Journal of Personality and Social Psychology, 86(2), 310–319. 10.1037/0022-3514.86.2.310 14769086

[R45] Wettstein M, Schilling OK, Reidick O, & Wahl H-W (2015). Four-year stability, change, and multidirectionality of well-being in very-old age. Psychology and Aging, 30(3), 500–516. 10.1037/pag0000037 26098169

[R46] Willroth EC, John OP, Biesanz JC, & Mauss IB (2020). Understanding short-term variability in life satisfaction: The Individual Differences in Evaluating Life Satisfaction (IDELS) model. Journal of Personality and Social Psychology, 119(1), 229–248. 10.1037/pspp0000261 31478706 PMC7050397

[R47] Wilms R, Lanwehr R, & Kastenmüller A (2020). Do we overestimate the within-variability? The impact of measurement error on intraclass coefficient estimation. Frontiers in Psychology, 825. 10.3389/fpsyg.2020.00825 PMC7248308 32508704

[R48] Yesavage JA, Brink TL, Rose TL, Lum O, Huang V, Adey M, & Leirer VO (1982). Development and validation of a geriatric depression screening scale: A preliminary report. Journal of Psychiatric Research, 17(1), 37–49. 10.1016/0022-3956(82)90033-4 7183759

