# Supplementary material for: Eudaemonic Well-Being in Midlife Women: Change in and Correspondence Between Concurrent and Retrospective Reports
Source: Collabra Psychol. Author manuscript; Available in PMC 2021 May 7. (PMC8104436; doi:10.1525/collabra.21433)
Supplement: Supp material [file NIHMS1690587-supplement-Supp_material.docx]

Eudaemonic Well-Being in Midlife Women: Correspondence between Daily and Retrospective Reports

Supplemental Online Material

**ZIP model for negative affect (NA) with Geriatric Depression Scale (GDS) symptom count as outcome.**

| *Term* | *Estimate* | *SE* | *p* | *Anti-log* |
| --- | --- | --- | --- | --- |
| **Zero generating process** |  |  |  | *Odds* |
| Intercept | -1.467 | 0.053 | < .001 |  |
| Mean diary NA | -0.234 | 0.016 | < .001 | 0.79 |
| Change in diary NA | -0.056 | 0.025 | .023 | 0.95 |
| **Symptom count** |  |  |  | *Count* |
| Intercept | 1.253 | 0.006 | < .001 | 3.5 |
| Mean diary NA | 0.052 | 0.001 | < .001 | 1.05 |
| Change in diary NA | 0.008 | 0.003 | 0.006 | 1.01 |

For each difference of +1 point in mean diary negative affect, the odds of a zero score go down by 21%, and if not zero, the predicted GDS symptom count increases by 1.05.

For each change of +1 point in diary negative affect across bursts, the odds of a zero score go down by 5%, and if not zero, the predicted GDS symptom count increases by 1.01.

**Correspondence results without perfect well-being: for example, if mean diary autonomy across all days = 100, then diary variables were replaced with NA. Compare Table 3 in the manuscript using all data.**

CI = 95% confidence interval; D = diary; random effect Person = person-level variance; random effect Person.change = random slope of A = autonomy, C = competence, R = relatedness, D = distress; random effect Person.Time = random slope of time (creates best-fitting autoregressive structure). The two ρ_01_ coefficients represent intercept-slope correlation for diary change slope and time slope, respectively.

**Reproducibility Information**

R version 3.6.1 (2019-07-05)

Platform: x86_64-w64-mingw32/x64 (64-bit)

Running under: Windows 10 x64 (build 18363)

Matrix products: default

locale:

[1] LC_COLLATE=English_United States.1252 LC_CTYPE=English_United States.1252 LC_MONETARY=English_United States.1252

[4] LC_NUMERIC=C LC_TIME=English_United States.1252

attached base packages:

[1] stats graphics grDevices utils datasets methods base

other attached packages:

[1] NCmisc_1.1.6 lavaan_0.6-7 misty_0.3.2 sjlabelled_1.1.1 sjPlot_2.7.2 dfoptim_2018.2-1 knitr_1.24

[8] lme4_1.1-21 Matrix_1.2-17 apaTables_2.0.5 psych_1.8.12 magrittr_1.5 ggplot2_3.3.2 dplyr_1.0.2

[15] plyr_1.8.4

loaded via a namespace (and not attached):

[1] ggrepel_0.8.1 Rcpp_1.0.2 mvtnorm_1.0-11 lattice_0.20-38 tidyr_1.0.0 assertthat_0.2.1

[7] digest_0.6.25 R6_2.4.1 backports_1.1.10 stats4_3.6.1 coda_0.19-3 highr_0.8

[13] pillar_1.4.6 rlang_0.4.7 rstudioapi_0.10 minqa_1.2.4 performance_0.3.0 nloptr_1.2.1

[19] pbivnorm_0.6.0 ggeffects_0.12.0 labeling_0.3 splines_3.6.1 foreign_0.8-71 munsell_0.5.0

[25] broom_0.7.0 compiler_3.6.1 modelr_0.1.5 xfun_0.8 pkgconfig_2.0.2 parameters_0.2.0

[31] mnormt_1.5-5 insight_0.9.6 tidyselect_1.1.0 tibble_3.0.3 fansi_0.4.0 crayon_1.3.4

[37] withr_2.1.2 MASS_7.3-51.4 sjmisc_2.8.2 grid_3.6.1 nlme_3.1-140 xtable_1.8-4

[43] gtable_0.3.0 lifecycle_0.2.0 git2r_0.26.1 bayestestR_0.7.2 scales_1.0.0 cli_2.0.2

[49] estimability_1.3 proftools_0.99-3 ellipsis_0.2.0.1 generics_0.0.2 vctrs_0.3.2 boot_1.3-22

[55] tools_3.6.1 forcats_0.4.0 glue_1.4.1 purrr_0.3.2 sjstats_0.17.5 hms_0.5.0

[61] emmeans_1.4 parallel_3.6.1 colorspace_1.4-1 haven_2.1.1

**R References**

Bache, S. M., & Wickham, H. (2014). *magrittr: A forward-pipe operator for r* [Manual]. https://CRAN.R-project.org/package=magrittr

Bates, D., Mächler, M., Bolker, B., & Walker, S. (2015). Fitting linear mixed-effects models using lme4. *Journal of Statistical Software*, *67*(1), 1–48. https://doi.org/10.18637/jss.v067.i01

Bates, D., & Maechler, M. (2019). *Matrix: Sparse and dense matrix classes and methods* [Manual]. https://CRAN.R-project.org/package=Matrix

Cooper, N. (2018). *NCmisc: Miscellaneous functions for creating adaptive functions and scripts* [Manual]. https://CRAN.R-project.org/package=NCmisc

Francois, R. (2017). *bibtex: Bibtex parser* [Manual]. https://CRAN.R-project.org/package=bibtex

Lüdecke, D. (2019a). *sjlabelled: Labelled data utility functions (version 1.1.1)* [Manual]. https://doi.org/10.5281/zenodo.1249215

Lüdecke, D. (2019b). *sjPlot: Data visualization for statistics in social science* [Manual]. https://doi.org/10.5281/zenodo.1308157

R Core Team. (2019). *R: A language and environment for statistical computing* [Manual]. https://www.R-project.org/

Revelle, W. (2018). *psych: Procedures for psychological, psychometric, and personality research* [Manual]. https://CRAN.R-project.org/package=psych

Varadhan, R., University, J. H., Borchers, H. W., & Research., A. C. (2018). *dfoptim: Derivative-free optimization* [Manual]. https://CRAN.R-project.org/package=dfoptim

Wickham, H. (2011). The split-apply-combine strategy for data analysis. *Journal of Statistical Software*, *40*(1), 1–29.

Wickham, H. (2016). *ggplot2: Elegant graphics for data analysis*. Springer-Verlag New York. https://ggplot2.tidyverse.org

Wickham, H., François, R., Henry, L., & Müller, K. (2020). *dplyr: A grammar of data manipulation* [Manual]. https://CRAN.R-project.org/package=dplyr

Xie, Y. (2014). knitr: A comprehensive tool for reproducible research in R. In V. Stodden, F. Leisch, & R. D. Peng (Eds.), *Implementing reproducible computational research*. Chapman and Hall/CRC. http://www.crcpress.com/product/isbn/9781466561595

Xie, Y. (2015). *Dynamic documents with R and knitr* (2nd ed.). Chapman and Hall/CRC. https://yihui.name/knitr/

Xie, Y. (2019). *knitr: A general-purpose package for dynamic report generation in r* [Manual]. https://yihui.name/knitr/

Yanagida, T. (2020). *Misty: Miscellaneous functions “t. Yanagida”* [Manual]. https://CRAN.R-project.org/package=misty
